# Adaptation of Melodic Intonation Therapy to Greek: A Clinical Study in Broca’s Aphasia With Brain Perfusion SPECT Validation

**DOI:** 10.3389/fnagi.2021.664581

**Published:** 2021-07-16

**Authors:** Maria Martzoukou, Anastasia Nousia, Grigorios Nasios, Spyridon Tsiouris

**Affiliations:** ^1^Department of Speech and Language Therapy, School of Health Sciences, University of Ioannina, Ioannina, Greece; ^2^Department of Nuclear Medicine, University Hospital of Ioannina, Ioannina, Greece

**Keywords:** Broca’s aphasia, melodic intonation therapy, non-invasive intervention, neural plasticity, brain perfusion SPECT

## Abstract

Melodic intonation therapy (MIT) is one of the most well-known treatment methods which is based on pitch and rhythm and was developed to increase verbal output in adults with non-fluent aphasia. Although MIT has been adapted to several languages, in Greece it is almost unknown. The aim of the proposed study is twofold: (1) to translate and adapt the MIT to the Greek language, and (2) to conduct an experimental study in order to examine the effect of MIT on Greek patients with Broca’s aphasia. To this aim, a 64-year-old, right-handed male who had a 6-year primary school education level, no musical abilities and poor performance on the recognition of prosody attended the MIT intervention program almost two and a half years after the event of suffering an ischemic stroke. The MIT intervention was administered three times per week for a 12-week period, in which each session lasted from 30 to 40 min. The patient underwent three assessments all using both the Boston Diagnostic Aphasia Examination–Short Form (BDAE-SF) and brain perfusion single-photon emission computed tomography (SPECT); (1) before the MIT, (2) immediately after, and (3) 3 months after the completion of MIT. The results from the BDAE-SF revealed an impressive improvement on both trained and prepositional speech production, immediately after the completion of the MIT, and a stable improved performance 3 months after MIT. The SPECT scan revealed reactivation of the perilesional areas of the left hemisphere, and considerably improved perfusion of the frontal lobe, the anterior temporal lobe, and the upper part of the parietal lobe of the right hemisphere. The improvement persisted and even expanded 3 months after MIT. Therefore, MIT is a promising intervention program and its positive effects last for at least 3 months after the completion of the intervention.

## Introduction

Aphasia is an acquired impairment in communication following brain damage (usually after a stroke), in most cases in the left hemisphere. Around 12% of the patients with aphasia are diagnosed as having Broca’s aphasia (Pedersen et al., 2004). Patients with Broca’s aphasia (non-fluent aphasia) are unable to speak fluently although their comprehension ability is well preserved. Apart from their difficulty in producing grammatical sentences (i.e., agrammatism), they might also experience word-retrieval difficulty (i.e., anomia), and a motor-speech disorder affecting the planning or programming of speech movements (i.e., apraxia of speech). According to Lazar et al. (2010) patients with Broca’s aphasia achieve 70% of the maximum possible recovery 3 months after the stroke, while two thirds of them continue to have language deficits at 18 months later, even after intensive speech language therapy (Laska et al., 2001). Thus, the effects of the traditional speech therapy treatment are relatively moderate (Turkeltaub, 2015).

For over 100 years, several clinicians and relatives of the patients reported that people with Broca’s aphasia are more capable of singing than of speaking the same words (Gerstman, 1964; Hébert et al., 2003; Racette et al., 2006). Music and prosody share the same cues, namely the variation of the pitch (intonation), the loudness (intensity), the duration, the tempo (rate) and the rhythm. These observations led to the creation of treatments involving either music or exaggerated prosody.

Melodic intonation therapy (MIT) (Albert et al., 1973; Sparks et al., 1974; Helm-Estabrooks et al., 1989) is one of the most well-known treatments that uses prosody, namely the musical element of speech, to improve expressive language in non-fluent aphasia. In particular, MIT is based on phrases produced with lengthened tempo, reduced variation of pitch, and exaggerated rhythm and stress, which leads to increased loudness (Sparks, 2008). Hand-tapping on the patient’s left hand according to the rhythm of the stimuli is considered as another essential characteristic of MIT. It has been claimed that it facilitates sound-motor mapping, offers continuous guidance for syllable production and can also engage the right-hemisphere sensorimotor network which controls hand as well as orofacial and articulatory movements (Helm-Estabrooks et al., 1989; Sparks, 2008; Norton et al., 2009). The goal of MIT is to help patients not only produce trained phrases, namely the phrases used during the treatment, but also to restore generative language to participants, thus making them able to produce propositional, non-trained language.

Even though many studies have reported the benefits of MIT in post-stroke aphasia [see also the systematic reviews by Hurkmans et al. (2012), van der Meulen et al. (2012), Zumbansen et al. (2014a), and Zumbansen and Tremblay (2019)], their results are not always comparable. For example, although hand-tapping is considered to be an important element in MIT, some studies applied modified versions of MIT in which no hand-tapping was used (Baker, 2000; Hough, 2010; Stahl et al., 2013), thus making their outcomes not directly comparable. Moreover, there are studies which do not provide any information regarding the musical background of their patients (e.g., Zumbansen et al., 2014b; Tabei et al., 2016; van de Sandt-Koenderman et al., 2018), although it has been argued that the impressive results reported in the case study conducted by Wilson et al. (2006) could be due to the patient’s better understanding of the prosodic and musical elements, given that he was an experienced musician (Zumbansen and Tremblay, 2019). Furthermore, unlike the goal of the original MIT, which is to restore generative language to participants, some studies used MIT only to train patients to produce a limited set of sentences, and they do not report any findings regarding patients’ ability to produce non-trained items (e.g., Goldfarb and Bader, 1979; Springer et al., 1993; Baker, 2000; Wilson et al., 2006; Hough, 2010). Lastly, although the duration of the positive effects of an intervention is of great importance, the long-term effects of MIT are not known. In particular, there is only one study (Wilson et al., 2006) reporting on patient’s performance some period (5 weeks) after the completion of the MIT intervention, but the results cannot be generalized, since the patient was a musician by profession.

As for the neural processes involved in MIT and the functional and structural changes that underlie the clinical results, they remain unclear as well. Although MIT was based on the assumption that the undamaged right hemisphere could take over the language functions of the damaged areas in the left hemisphere (Albert et al., 1973; Sparks et al., 1974), this hypothesis was not always supported by neuroimaging studies. In particular, some studies reported increased activity of the right hemisphere after the completion of MIT (Schlaug et al., 2008, 2009; Zipse et al., 2012), while others failed to find any clear evidence regarding right hemisphere activation (Laine et al., 1994; van de Sandt-Koenderman et al., 2018). On the other hand, several studies related improved expressive language abilities to a decreased activation of the right hemisphere, compared to the baseline before treatment (Wan et al., 2014; Tabei et al., 2016), or to an increased activation of the perilesional areas of the left hemisphere (Belin et al., 1996; Breier et al., 2010). The reason for these inconsistencies could be due to differences among the participants (severity and exact location of the lesion), to differences regarding the time of administration of MIT after stroke and to the length of the therapy, as well as due to differences on the imaging modality and the activation task used [e.g., functional magnetic resonance imaging (fMRI) (Schlaug et al., 2008; Zipse et al., 2012; Tabei et al., 2016; van de Sandt-Koenderman et al., 2018); magnetoencephalography (MEG) (Breier et al., 2010); positron emission tomography (PET) (Belin et al., 1996); and single-photon emission computed tomography (SPECT) (Laine et al., 1994)].

Therefore, it is clear that more systematic research on the use of a neuroimaging technique is needed, in order for reliable conclusions to be drawn regarding the effectiveness of MIT. In particular, studies should offer clear descriptions about patients’ understanding of the musical/prosodic cues, based on their musical (or not) background (e.g., Wilson et al., 2006) or on their performance on relevant tests, before the administration of MIT, and clear results about their ability to produce both trained and non-trained items, after the completion of MIT.

Although MIT has been adapted to several languages and cultures (other than American English), such as Japanese (Seki and Sugishita, 1983), French (van Eeckhout and Bhatt, 1984), Persian (Bonakdarpour et al., 2003), Brazilian Portuguese (da Fontoura et al., 2014), Italian (Cortese et al., 2015), and Spanish (Haro-Martínez et al., 2017), it has not been adapted to conduct research on Greek and its use in Greece is practically non-existent. It is necessary to adapt MIT and not just to translate it literally, since not all languages share the same prosodic properties (tone, rhythm, order of stressed and unstressed syllables). Moreover, the effectiveness of the first stage of MIT is based on the use of formulaic phrases. Thus, phrases which are commonly used in each specific language are needed (Zumbansen et al., 2014a). Lastly, in order to ensure similarity between the adapted version and the original English one, the feasibility of each MIT language adaptation should be tested in stroke patients who have ended up with non-fluent aphasia (Helm-Estabrooks et al., 1989).

Therefore, the aim of the present study is twofold: (a) to translate and adapt MIT (Albert et al., 1973; Sparks et al., 1974; Helm-Estabrooks et al., 1989) to Greek, and (b) to examine its efficiency in Broca’s aphasia patients, through the use of neuropsychological batteries [Boston Diagnostic Aphasia Examination (BDAE); Goodglass et al., 2001] and neuroimaging techniques [single-photon emission computed tomography (SPECT)].

## Materials and Methods

### Adaptation of MIT

Based on descriptions by Helm-Estabrooks et al. (1989), a set of formulaic phrases of suitable length and increasing difficulty was developed. In particular, three levels with 20 items in each level were prepared. Phrases which sounded strange in Greek were replaced by more commonly used Greek phrases, or by phrases that are necessary in the daily life of the patient. Greek is a language with rich morphology, thus most words and phrases have more than two syllables. For this reason, in contrast to the American English version of MIT which starts with 2 to 3 syllable phrases, the first level of the Greek MIT contained phrases from 2 to 4 syllables, the second up to six syllables, while the final level also contained phrases of more than six syllables.

As for the prosodic cues, phrases were produced by the experimenter with lengthened tempo, exaggerated rhythm and stress, and reduced pitch variation. In particular, they were intoned on two pitches separated by a perfect 4th, with the stressed syllables to be produced on the higher pitch and the unaccented syllables on the lower pitch of the two. Greek is trochaic language, in which a stressed syllable is followed by an unstressed one. Moreover, it is a language with dynamic stress and follows the trisyllabic constraint which requires the stress to fall only in the region up to three syllables from the end of the word or phrase. The experimenter took into consideration the aforementioned characteristics and after practicing speaking her items, she made sure that all phrases were produced with the use of these tones.

Helm-Estabrooks et al. (1989) MIT kit contains 60 action picture cards depicting the practice phrases, in order for the auditory stimuli to be reinforced. For our patient, however, these pictures proved to be confusing and distracting, probably due to their style of drawing which is alien to the old Greek culture, or, as Sparks (2008) supports, due to the fact that MIT candidates have a good auditory comprehension and, thus, do not need added stimuli. For that reason, no pictures were used in the first two levels, whereas pictures depicting familiar objects from the participant’s daily life (e.g., a cup of Greek coffee) were used in the final level to trigger him to initiate a relevant sentence on his own and not to repeat whole or part of the experimenter’s utterance.

### Pilot Intervention

#### Patients

The speech-therapist of our team (A.N.) checked her records for patients she had treated at least 2 years earlier and together with the primary investigator selected four patients as being suitable for the MIT intervention. The criteria for the participants’ selection were: to be chronic patients of Broca’s aphasia (at least 2 years after stroke), to have suffered only one, unilateral, left-hemisphere stroke, to present severely restricted speech output and bad repetition of sentences but well preserved auditory comprehension ability, and to have a relatively good emotional state and motivation. After contacting the patients to discuss the research study, one did not consent to participating, while the rest of them agreed to be enrolled. All three patients and their families were orally and written informed about the purpose and the exact procedure of the intervention and they signed consent forms according to the Declaration of Helsinki. Two of the three patients, though, interrupted the intervention; one due to the fact that she suffered a second stroke just before the completion of the intervention, while the other found the intervention program too intensive to follow (restrictions of time and transportation). Therefore, only one patient completed the MIT intervention program; a 64-year-old, right-handed male (we termed him “Th.G.”) who had completed a 6-year primary school education background.

The participant had suffered an ischemic stroke in July 2016, 2 years and 7 months before the beginning of the MIT intervention. In January 2017 he was admitted to a private rehabilitation center for a year’s care. During his sojourn, he received services by a clinical team consisting of psychologists, physiotherapists, occupational therapists, and speech therapists. He attended the traditional speech therapy intervention, administered by the second author for 16 months (until April 2018), which had consisted of 30- to 40-min sessions, 3–4 times per week. His clinical state at the beginning of the conventional speech therapy intervention was: use of nasogastric tube feeding, total inability to speak (Broca’s aphasia), good comprehension ability, ability to follow simple and complex orders. The intervention consisted of swallowing exercises (e.g., Shaker, Masaco), myofunctional exercises, oral-motor and laryngeal massage, verbal apraxia intervention program using the Sings Make Talk (SIMATA) method, and practice of common sequences (e.g., days of the week), everyday words (e.g., water, food) and automatic phrases (e.g., how are you?).

A month after the beginning of the traditional speech therapy treatment, the patient was able to swallow and eat on his own. Throughout the conventional therapeutic program, however, the improvement in his expressive language ability was limited and did not improve much after the initial sessions. Thus, it was considered that his verbal output had reached a plateau. In particular, Th.G. became able to use automatic one-word phrases and to communicate with the use of gestures and yes/no replies. When he left the private clinic he returned to his village and his family, where he continued to spend at least 4 h each day with his friends, as he used to do before the stroke. This return to his usual life helped him to reinforce his emotional stability.

At the beginning of the MIT intervention (March 2019), Th.G. had just completed four extra months of speech-language therapy which he attended once a week, but without any further improvement. His language abilities were evaluated with the use of the four–out of the total number of five–sections of the Boston Diagnostic Aphasia Examination–Short Form (BDAE-SF) [Goodglass et al. (2001), for Greek see Messinis et al. (2013)]. In the first subsection (Simple social responses) of the first functional section (I. Conversational and expository speech), he answered with automatic one-word phrases: “kalispera” (good evening), “kala” (fine), “ne” (yes), and “ohi” (no). When he was asked to produce his full name, although he understood the question, he pronounced only his first name. In the next subsection (Free conversation) he was unable to start a conversation on his own, while he found difficulty in talking about any of the discussion topics suggested by the speech-therapist (e.g., presentation of himself, description of his family, description of his daily routine, description of the clothes he was wearing). In the last subsection (Picture description), he did not manage to supply any description [“eee… ohi (no)…ohi (no)…eee”]. In the next functional section (II. Auditory comprehension), the examination revealed good comprehension abilities, with only some isolated comprehension disturbances when phrases were syntactically complex. Regarding language production (III. Oral expression), Th.G.’s performance showed dramatically reduced ability, severe diminution of speech initiation and verbal output restricted to one-word replies and automatic phrases. His repetition ability was found to be good at word level, but severely impaired at the sentence level. No paraphasias were found. Lastly, the patient’s reading ability (IV. Reading) was intact at letter and number recognition but it would get worse as the difficulty of the subsections was raised (reading of words or sentences and reading with comprehension).

Regarding the participant’s musical abilities, he could not play any musical instrument. In order to further examine his ability to comprehend prosodic cues, Affective Prosody Test (APT) (Kosmidis and Vlahou, 2001) was administered. APT consists of five neutral content sentences, audio-recorded while produced by an actor with prosody expressing anger, fear, happiness, sadness, surprise and neutrality. Th.G.’s performance was compared to that of an age-matched control group consisting of 20 community-dwelling healthy participants. In particular, the control group consisted of 10 females and 10 males, with an age range from 61 to 72 years old, a mean age of 69.2 years, a range of years of education from 6 to 12 years and a mean educational level of 8 years and 7 months (see Table 1).

**TABLE 1:** Mean percent (%) responses of the control group (CG) and our patient (Th.G.) on the APT.

	Happiness	Anger	Sadness	Fear	Surprise	Neutrality	Overall
**Th.G.**	40	20	20	60	40	20	33.3
**CG**	76	46	69	63	61	84	66.5

Despite the participant’s limited expressive language abilities and poor performance on APT, when his favorite song was played by a professional accordionist, he managed to sing along some of the lyrics. Lastly, neurological examination revealed right-sided hemiparesis, without any hearing deficit.

Therefore, the main characteristics of the patient under investigation were the following: (a) unilateral, left-hemisphere stroke, (b) severely restricted speech output, (c) ability to produce several lyrics while singing favorite songs, (d) relatively good repetition of single words, but severely impaired at the sentence level, (e) well preserved auditory comprehension, and (f) emotional stability and good motivation. The combination of these characteristics is considered to be “ideal” for a candidate to undergo MIT (Sparks, 2008).

#### MIT Protocol

Before the initiation of the MIT intervention, the primary investigator had two meetings with the patient. In the first one, she explained to the patient and his family the exact purpose and procedure of the MIT program and asked for his consent to participate in the study. Moreover, she collected information regarding the patient’s daily routine, family relationships, and personal likes and dislikes, and encouraged his family to help with the selection of the most useful phrases for their communication. In the second meeting, she administered the APT (Kosmidis and Vlahou, 2001) to the patient and recorded the lyrics produced by him while listening to his favorite song being played by a professional accordionist. Apart from evaluating the patient’s language production and prosodic cues comprehension ability, these two meetings served to establish a sense of safety, assertiveness and hopefulness, and a positive therapeutic relationship between the investigator and the patient. Moreover, the material used during the treatment was modified based on the information collected from the family regarding the necessities in the daily life of the patient.

After the first meeting and before the beginning of the MIT intervention, the patient’s clinical and medical condition was also evaluated by the experienced neurologist of our team (G.N.) and by his speech-therapist (A.N.) with the use of the BDAE-SF and then he was referred for the baseline SPECT scan.

The MIT intervention was administered three times per week for a 12-week period (25th of February 2019 to 19th of May 2019). Each session lasted around 30–40 min. MIT program was divided into four levels, as was the case in the original one (Albert et al., 1973; Sparks et al., 1974; Sparks, 2008). The first level is the non-verbal one. The clinician hums the intonation of the target words or phrases and signals the patient to join her/him in unison humming. During this level, the patient is introduced to melodic intonation, hand-tapping and to the hand-signals controls of the clinician. In the next three levels, linguistic material is added and participants are required to repeat the intoned phrases. Linguistic material of progressive difficulty is used; from easily articulated words and phrases consisting of 2–4 syllables used in the second level, to more phonologically complex phrases of more than six syllables in the last level. The difficulty in level III is further enhanced by progressively reducing the clinician’s participation, while at the final step of the same level, patients are required to respond to short intoned questions related to elements of the presented phrases. At level IV, longer delays are imposed before the clinician allows the patient to repeat the phrases, whereas phrases are now presented by the clinician firstly with exaggerated prosody (sprechgesang technique) and then in normal speech. Questions at this level are related to more substantive information contained in the practicing phrase.

Each level consisted of 20 items in which the difficulty increased progressively. At the end of each session, the participant’s performance was calculated on a percentages basis, namely the number of items successfully completed out of the total of 20 items. When the patient accomplished a score higher than 95% for five consecutive sessions, the next level was administered (Helm-Estabrooks et al., 1989). Hand-tapping on the patient’s left hand according to the rhythm of the stimuli was used throughout the intervention (Helm-Estabrooks et al., 1989).

**FIGURE 1:**
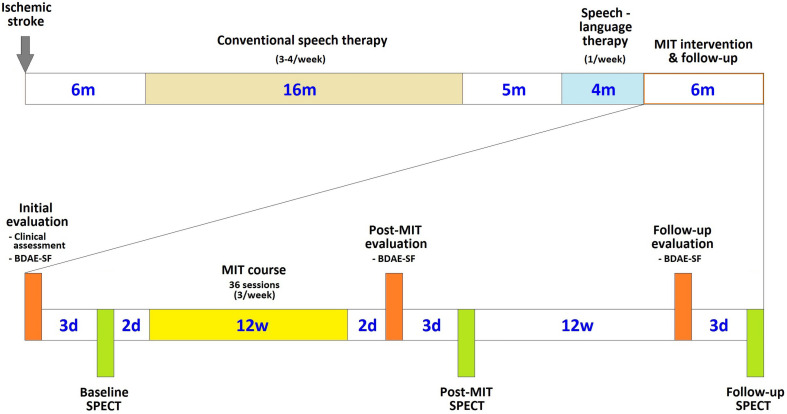
Chronological arrangement of the MIT course and its associated clinical and language assessments and imaging sessions in relation to the patient’s stroke and subsequent therapeutic interventions (d is for days, w for weeks, and m for months).

The patient underwent three language assessments with the use of BDAE-SF, along with SPECT scanning in close temporal vicinity (within 1–3 days) to the language assessment: (1) before starting the 12-week period of MIT intervention, (2) immediately after the completion of the treatment, and (3) 3 months later (Figure 1).

These three evaluations offered us a clear view of the effects of the therapeutic intervention and the duration of any positive effects.

#### Brain Perfusion SPECT Protocol

Brain perfusion SPECT was chosen as the imaging method to visualize any deficits in cortical perfusion and neuronal activity, as well as for depicting potential improvements in cortical function ensuing from the MIT intervention. The principle of this modality lies in the tight physiologic coupling between cortical blood flow and metabolism, in order to secure adequate oxygenation and glucose supply to the neurons (Miller and Bell, 1987). Before the scan, the patient was meticulously informed about the procedure and consented to it, involving the catheterization of an antecubital vein in the forearm and the intravenous administration of 740 MBq activity of the lipophilic radiopharmaceutical Technetium-99m hexamethyl propylene amine oxime (^99m^Tc-HMPAO; Ceretec, GE Healthcare AS, Oslo, Norway). The radiotracer was labeled in-house according to the manufacturer’s preparation instructions. In order to minimize any external stimuli to the brain that could interfere with cortical activity and affect cerebral perfusion and image interpretation, the radiotracer injection was carried out with the patient resting supine and relaxed in a dimly lit, quiet room for 10 min.

Imaging was initiated 30 min post-injection in a dual-detector γ-camera (Optima 640 NM CT, GE Healthcare, Chicago, IL, United States), equipped with a pair of low-energy, high-resolution, parallel-hole collimators. The patient was supine on the camera bed and his head was held with fixation strips attached to a dedicatedly constructed head holder, thus allowing the camera detectors to rotate very near to the head. Data were acquired over one full rotation in step-and-shoot mode (3° angular step, 128 projections, 30 s/projection, total study duration 32 min); the matrix resolution was 128 × 128 pixels, with the photopeak centered at 140 keV and a symmetrical 10% window. After completion of raw data acquisition, this was inspected in cine mode for possible sources of error (mostly occurring from patient movement), followed by image reconstruction processing with the use of the filtered back-projection algorithm (Butterworth filter with critical frequency 0.5 and power 10; Chang attenuation correction method with threshold 10 and cut-off 0.11), producing cortical perfusion tomographic images in the three planes (transversal, coronal and sagittal).

The 3-dimensional (3D) tomographic images were first assessed qualitatively in the transversal plane. Then, image analysis was performed on the reconstructed data with the use of the dedicated software NeuroGam (Segami Corporation, Columbia, MD, United States). This incorporates the brain SPECT imaging data of a reference control group of normal individuals and allows the semiquantitative evaluation of patient brain perfusion in specific Brodmann Areas (BA) of the cortex. The control group consists of 64 subjects equally split between the two genders and age-stratified. During semiquantitative analysis, the mean regional cortical perfusion score of normal controls of the same age and gender serve as a comparison reference for the recorded perfusion score of studied patients.

## Expected Clinical Benefits

The expected results after the completion of the MIT program are for patients to be able to communicate by using suitable phrases for every occasion, even if their speech is articulated poorly and in a telegraphic manner (Sparks, 2008). The term “telegraphic” indicates the existence of verbs, nouns, adjectives, and adverbs in patient’s sentences but the omission of functional words (e.g., articles, prepositions, connectives, etc.) that do not add any information to the meaning of the phrase but help the utterance to be grammatically correct.

Based on the main aim of the original MIT (Albert et al., 1973; Sparks et al., 1974), according to which patients, after the completion of the program, should not only be able to produce phrases that have been used during the intervention, but also to generate their own, new phrases, Th.G. is expected to be able to use propositional, non-trained language, apart from automatic, formulaic phrases.

Although not many studies have explored or commented about the duration of the positive effects of MIT on language production, ideally any improvement should remain long after the completion of the intervention. Therefore, we expect any achieved language improvement to be retained by Th.G. until our next programmed evaluation of his language ability, that is 3 months after the MIT intervention. According to Sparks (2008), however, MIT graduates are expected not only to maintain their language production achievements but also to continue to improve their language expression ability in their own home environment.

Regarding brain activity, even though MIT predicts the undamaged right hemisphere to take over the language functions of the damaged areas in the left hemisphere (Albert et al., 1973; Sparks et al., 1974), this hypothesis has not always been supported by neuroimaging studies, and, thus, no clear assumptions can be made.

## Results

### Language Assessment

#### Language Assessment After MIT

The language abilities evaluation, just after the completion of MIT, showed improved results in all four tested functional sections of BDAE-SF. In particular, in the subsection “Simple social responses” of the first functional section, “I. Conversational and expository speech,” our patient supplied answers to all the questions made. In the next subsection, “Free conversation,” he was asked to describe how he spends his day and he answered: “ksipnao tis okto ce misi… plenome… skupizome…eee… pigeno sto trapezi… troo to tost. eee… kafenio pino kafe…meta tsipuro… hahaha… meta pao spiti” [I wake up at half past eight… I am washed (I wash my face)… I am wiped (I wipe my face)…ehh… I go to the table… I eat toast… ehh… (at the) coffeehouse I drink coffee… then tsipouro (a traditional Greek brandy spirit)… (sniggers because he is not allowed to drink alcohol)… then I go home]. In the last subsection, “Picture description,” he described “The cookie theft” picture as follows: “dio pedjia… to pedi pai na pesi… eee… i mama skupizi ta phiata… oh. oh… ta nera trehun” [Two children… the child is ready to fall… eeh… the mother is wiping the dishes… Oh! Oh! (exclamations of dissatisfaction) the water is running].

Improvement was also obvious in the remaining functional sections (see also Table 2). Taking into consideration that the difficulty of the items used in each subsection of the BDAE-SF raises gradually, Th.G., after the completion of the MIT, managed to follow all the commands, even the final one which consists of five different steps, and to answer to all questions, even the ones related to the last (and more demanding) short passage of the subsection “Complex ideational material.” The patient’s improvement, however, was more pronounced in the section “Oral expression.” His ability to produce automatized sequences had improved and he became able to repeat whole sentences (“Repetition of sentences”) and not only isolated words. The greatest improvement, though, was found in his ability to name the items requested by relevant oral descriptions (“Responsive naming”) or depicted on pictures (“Boston naming test–short form,” “Special categories screening”).

**TABLE 2:** Th.G.’s performance on each subsection of the three sections of BDAE-SF before (1st assessment), just after MIT (2nd assessment), and 3 months after the completion of MIT (3rd assessment) (* indicate significantly low performance, bolded numbers indicate significant progress).

BDAE-SF		1st assessment	2nd assessment	3rd assessment
II. Auditory comprehension	Word comprehension	16/16	16/16	16/16
	Commands	7/10	**10/10**	9/10
	Complex ideational material	3/6	**6/6**	5/6
III. Oral expression	Automatized sequences	2/4	**4/4**	4/4
	Repetition of words	4/5	4/5	5/5
	Repetition of sentences	*0/2	**2/2**	2/2
	Responsive naming	*0/10	**9/10**	10/10
	Boston naming test (short form)	*1/15	**10/15**	**14/15**
	Special categories screening	*1/12	**7/12**	6/12
IV. Reading	Letter and number recognition	8/8	8/8	8/8
	Picture-word matching	3/4	**4/4**	4/4
	Word reading	*3/15	**14/15**	14/15
	Reading of sentences	*0/5	**2/5**	2/5
	Reading of sentences with comprehension	*0/3	**3/3**	3/3
	Comprehension of sentences and paragraph	2/4	**4/4**	3/4

Lastly, the patient’s performance on “Reading” was also better, probably due to his improved ability to produce language. In particular, his performance was better when compared to the first evaluation in the “Word reading” subsection, in which participants are asked to read five words as quickly as possible (score changes according to the participant’s reaction time–3 is the highest score per item). It appears that the participant became able to read more words within the shortest time (0–3 m/s). Moreover, his performance improved in “Picture-word matching,” “Reading of sentences,” “Reading of sentences with comprehension,” “Comprehension of sentences and paragraph” subsections, in which participants are requested to read and comprehend words, sentences and paragraphs.

Moreover, Th.G. became able to use the trained items (e.g., “my stomach hurts,” “go to the hospital”) in different contexts. For example, on his own initiative, he informed the experimenter that one of his friends was not at the coffeehouse the previous day, because his stomach was in pain and he had gone to the hospital. Interestingly, though, his ability to produce language also exceeded the learned items. For example, during the last sessions, in which the experimenter asked him the reason he was not wearing his watch, he said “halase i bataria… piga na to ftiaksun… tin epomeni evdomada” [the battery died… I took (it) to have it fixed… next week (it will be ready)]. Similarly, he informed the experimenter that his favorite coffeehouse was about to close for a week due to renovations and that he had been invited to the wedding of his best friend’s daughter which was going to be held in August. His utterances did not exceed the use of 4–5 words per utterance, and functional words (e.g., articles, pronouns, and prepositions) were included in several mandatory language environments. According to his family, the patient began to produce more complete and appropriate utterances outside the therapy room as well. Thus, although at the beginning Th.G. was skeptical about the outcomes of the MIT, due to the long time that had elapsed since the stroke, the limited results of the previous interventions and the “amusing” procedure of the MIT, as he characterized it, he seemed to have been happily surprised about his progress after the completion of the intervention. His initial motivation, namely to be able to better communicate with his friends and family about his daily needs, surpassed his will to initiate conversations and provide information about subjects other than his needs.

#### Language Assessment 3 Months After MIT

Three months after the completion of MIT, our patient underwent a BDAE-SF language assessment. He was still able to supply an answer to all the questions addressed to him, while his descriptions regarding the way he spends a day [“ksipnao… troo…eee… meta volta… kafenio pino kafe…eee… pao spiti–(I wake up… I eat… ehh… then cafeteria I drink coffee… ehh… I go home)] or the “The Cookie Theft” picture [“pedjia… pefti… nera down”–[children… (it) falls… waters down)] were simpler compared to his performance immediately after the completion of MIT. In the remaining functional sections, Th.G. demonstrated a, relatively, stable ability (see Table 2).

The patient did not undergo any speech therapy treatment from the time of completion of MIT until this last language assessment. Moreover, as we were informed by the patient’s family members, although they tried to help him revise the final steps of MIT (as they were advised to do by the experimenter), he was not cooperative. Lastly, as the family claimed, during the treatment and just after the completion of MIT, he was trying to form complete phrases. In the months that followed, though, he started to make more effort to use more new words rather than complete phrases, since “he felt that everybody could understand him anyway,” as his family members said.

### Brain Perfusion SPECT Scan at Baseline, Immediately After MIT, and at the Time of the 3-Month Follow-Up

**FIGURE 2:**
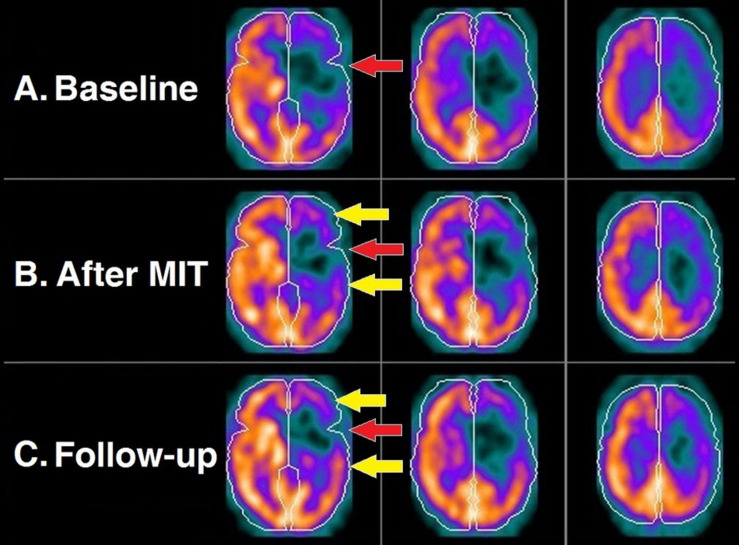
Brain perfusion scintigraphy at the three time points of the study: before MIT **(A)**; right after its completion **(B)**; and 3 months later **(C)**. Each row depicts three consecutive transversal SPECT slices of the brain. Perceptible radiotracer uptake (warm colors) represents cortical areas with normal blood supply, as opposed to those exhibiting diminished to negligible uptake (cold colors) denoting mildly to severely compromised perfusion. Expectedly, the necrotic core of the stroke (*red arrow*) remains unaltered in all three scans. However, there is an evident gradual perfusion improvement of the hypoperfused cortex surrounding the necrotic core (*yellow arrows*).

The first assessment of the patient before the MIT intervention (baseline brain perfusion scan) revealed findings consistent with the patient’s history of stroke. There was profoundly diminished uptake of the radiotracer primarily at the anatomical bed of the left middle cerebral artery, a finding consistent with impaired blood supply in the corresponding cortical area (Figure 2A). The central area of this cortical perfusion deficit was prominent also on the scintigraphic assessment conducted immediately after the completion of the MIT, an expected finding since it corresponded to the necrotic core of the stroke; nevertheless, an improvement in cortical perfusion was also revealed at the periphery of the deficit (Figure 2B). On follow-up 3 months after MIT, this peripheral perfusion gain became quite apparent (Figure 2C).

**FIGURE 3:**
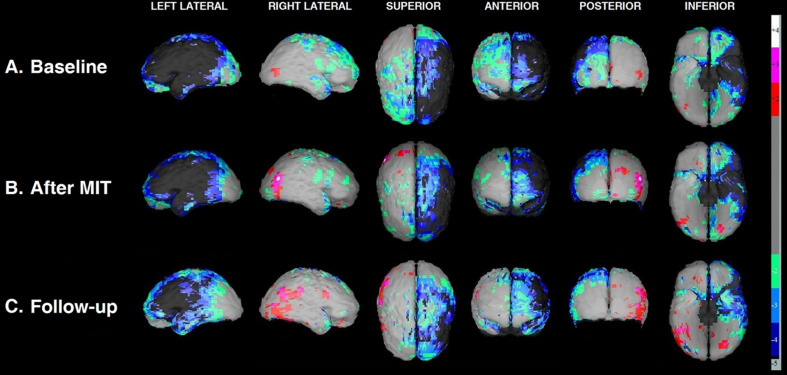
Three-dimensional display of the cortical blood flow by NeuroGam in various views at the three time points of the study: before MIT **(A)**; right after its completion **(B)**; and 3 months later **(C)**. Cortical perfusion is depicted as the difference between the patient’s perfusion score and the mean score of age- and gender-matched reference controls and is expressed as a fraction of the control’s SD value (henceforth referred to as *Z*-score). Cortical areas with scores laying in the range between –1.99 SD and +1,99 SD (i.e., regions imaged in light gray) are considered normal to marginally hypo- or hyperperfused, respectively, hence the software does not assign to these any particular color coding. Those areas that exhibit significant to severe hypoperfusion (*Z*-scores from –2.0 SD to –5.0 SD) are color-coded cold (green, light blue, dark blue, and dark gray), while the substantially hyperperfused ones (*Z*-scores from +2.0 SD to +5.0 SD) are color-coded warm (red, magenta, and white).

The improvement, from the baseline SPECT scan to that immediately after the MIT and then at the 3-month follow-up, is easily perceptible on the semiquantitative cortical perfusion analysis by NeuroGam (Figure 3). At baseline (Figure 3A), the hypoperfused areas occupied the left frontal lobe almost entirely and extended to a considerable proportion of the left parietal and temporal lobes. Furthermore, certain hypoperfused areas were revealed on the right hemisphere, situated on the frontal lobe, the upper portion of the parietal lobe and the anterior part of the temporal lobe. Immediately after the MIT intervention (Figure 3B), there was a borderline perfusion improvement at the periphery of the deficit, which was maximized and became quite noticeable 3 months later (Figure 3C), leading to a clear reduction of the hypoperfused cortical areas of the left hemisphere.

Table 3 summarizes the cortical BA perfusion scores calculated by NeuroGam in the three scans; these scores form the basis of the 3D perfusion images depicted in Figure 3. In every BA, the software compares the patient’s radiotracer uptake with that of age- and gender-matched controls and calculates a *Z*-score, which represents the difference between the patient’s score and the controls’ mean value in the corresponding BA as a fraction of the controls’ SD value. A perfusion *Z*-score of 0.0 SD suggests no deviation from the controls’ mean, with positive values representing hyperperfusion and negative ones denoting hypoperfusion. Any significant *Z*-score increase between the two scans is considered to represent an improvement in cortical perfusion and, correspondingly, in neuronal activity.

**TABLE 3:** Cortical perfusion mapping of the patient compared against NeuroGam’s database of age- and gender-matched normal controls in the three time points of the study.

Brodmann area	Left hemisphere Z-scores					Right hemisphere Z-scores				
	Baseline	After MIT	Follow-up	1st vs. 2nd scan	2nd vs. 3rd scan	Baseline	After MIT	Follow-up	1st vs. 2rd scan	2nd vs. 3rd scan
*4*	**−4.7**	**−4.3**	**−3.6**	0.4	0.7	**−2.2**	−1.4	−0.7	0.8	0.7
*5*	**−4.7**	**−4.3**	**−4.0**	0.4	0.3	−1.9	0.1	−1.1	**2.0**	−1.2
*6*	**−4.6**	**−4.4**	**−3.8**	0.2	0.6	**−2.3**	−1.3	−1.2	**1.0**	0.1
*7*	**−4.2**	**−3.5**	**−2.7**	0.7	0.8	−0.7	0.5	0.6	**1.2**	0.1
*8*	**−4.9**	**−4.8**	**−4.6**	0.1	0.2	**−2.8**	−1.6	−1.5	**1.2**	0.1
*9*	**−4.9**	**−4.7**	**−4.2**	0.2	0.5	**−2.3**	−1.7	−0.4	0.6	**1.3**
*10*	**−4.5**	**−3.9**	**−3.3**	0.6	0.6	**−2.2**	−1.3	−0.9	0.9	0.4
*11*	**−2.8**	**−2.8**	−1.7	0.0	**1.1**	−1.3	0.0	−0.4	**1.3**	−0.4
*12*	**−3.7**	**−4.3**	**−4.0**	−0.6	0.3	**−2.5**	**−2.6**	**−2.3**	−0.1	0.3
*17*	−1.2	−0.4	0.6	0.8	**1.0**	0.4	−0.1	0.6	−0.5	0.7
*18*	−1.4	−0.6	0.1	0.8	0.7	0.3	−0.5	0.3	−0.8	0.8
*19*	**−2.0**	−1.0	0.0	**1.0**	**1.0**	0.5	0.9	0.8	0.4	−0.1
*20*	**−4.9**	**−4.7**	**−3.5**	0.2	**1.2**	−1.1	−0.6	0.1	0.5	0.7
*21*	**−4.9**	**−4.6**	**−3.8**	0.3	0.8	−1.0	−0.2	0.2	0.8	0.4
*22*	**−5.0**	**−5.0**	**−4.5**	0.0	0.5	−1.4	−0.2	−0.5	**1.2**	−0.3
*23*	**−3.6**	**−3.7**	−1.0	−0.1	**2.7**	−1.8	−1.9	0.1	−0.1	**2.0**
*24*	**−2.5**	**−2.3**	**−2.2**	0.2	0.1	**−2.8**	**−3.2**	−1.8	−0.4	**1.4**
*25*	**−2.9**	**−3.0**	**−3.0**	−0.1	0.0	**−2.7**	**−2.8**	**−2.7**	−0.1	0.1
*28*	**−4.5**	**−4.5**	**−3.6**	0.0	0.9	**−3.1**	**−2.4**	−0.8	0.7	**1.6**
*31*	**−3.8**	**−2.9**	−1.7	0.9	**1.2**	−1.2	−0.7	0.2	0.5	0.9
*32*	**−4.1**	**−3.5**	**−2.7**	0.6	0.8	**−2.8**	**−2.1**	−1.4	0.7	0.7
*36*	**−4.6**	**−4.9**	**−3.5**	−0.3	**1.4**	−1.5	−1.7	−0.1	−0.2	**1.6**
*37*	**−4.6**	**−4.4**	**−2.5**	0.2	**1.9**	−0.3	0.1	1.5	0.4	**1.4**
*38*	**−5.0**	**−4.9**	**−4.1**	0.1	0.8	**−2.5**	**−2.5**	**−2.1**	0.0	0.4
*39*	**−4.7**	**−4.2**	**−2.2**	0.5	**2.0**	−0.1	0.9	**2.2**	**1.0**	**1.3**
*40*	**−5.0**	**−5.0**	**−3.9**	0.0	**1.1**	−0.3	−0.1	1.5	0.2	**1.6**
*44*	**−5.0**	**−5.0**	**−5.0**	0.0	0.0	−1.4	−1.0	−0.3	0.4	0.7
*45*	**−5.0**	**−4.8**	**−5.0**	0.2	−0.2	−1.3	0.1	0.3	**1.4**	0.2
*46*	**−5.0**	**−4.9**	**−4.6**	0.1	0.3	−1.7	−0.9	0.1	0.8	**1.0**
*47*	**−4.0**	**−3.9**	**−3.2**	0.1	0.7	−0.4	0.0	0.0	0.4	0.0

*Scores exceeding the range ±2.0 SD (marked in bold) are considered indicative of clinically significant hyper- or hypoperfusion, respectively, and the same applies for changes in perfusion between two scans exceeding ±1.0 SD (also marked in bold).*

As depicted in Table 3, on the left hemisphere, the Broca area (BA 44 and 45) remained heavily hypoperfused in all three scans, which denotes that it is practically necrotic, since it is situated at the core of the stroke infarct. At the periphery of the extensively hypoperfused infarcted area, however, there is a wide area of marked hypoperfusion that exhibited a borderline perfusion improvement already noticeable between the baseline and the post-MIT scan (mostly BA 19), with this peripheral perfusion gain becoming even more apparent and readily discernable in the third (follow-up) scan at 3 months. The cortical regions exhibiting improved perfusion following the MIT pertain to rear and middle sections of the parietal lobe (BA 23, 31, 39, and 40), the temporal lobe (BA 20, 36, and 37), the anterior part of the occipital lobe (BA 19) and, to a lesser degree, the frontal lobe (BA 11). T he perfusion of the Wernicke area–comprising BA 22 and adjacent parts of the heteromodal cortex (BA 39 and 40) (Mesulam, 1998)–was slightly improved in BA 22, but it was markedly improved in the areas BA 39 and 40, with the most significant improvement occurring between the second and third scan.

Concerning the areas of reduced cortical perfusion of the right hemisphere, most of them improved or even normalized already during the post-MIT scan (e.g., BA 4, 6, 8, 9, and 10 on the frontal lobe and BA 5 on the parietal lobe), with further improvement occurring in the 3-month follow-up in the frontal lobe (BA 9) and the temporal lobe (BA 28 and 38). As for the right-sided Wernicke’s area, BA 22 exhibited a perfusion increase similar to that of the ipsilateral (right-sided) Broca’s area; what is most striking, however, is that its other two regions (BA 39 and 40) gradually increased from normal at baseline to hyperperfused at 3 months after MIT (particularly BA 39).

## Discussion

A 64-year-old, right-handed male (Th.G.) at a 6-year primary school education level, no musical abilities and poor performance on the recognition of prosody, as it was measured with the use of APT (Kosmidis and Vlahou, 2001) attended the MIT intervention program almost two and a half years after the occurrence of an ischemic stroke of the left hemisphere. The MIT intervention was administered three times per week for a 12-week period, with each session lasting from 30 to 40 min. The patient underwent three assessments with the use of BDAE-SF and SPECT; the first one before the MIT, the second one immediately after it and the third 3 months after the completion of MIT.

### Baseline Measures

According to the “Auditory comprehension” section of BDAE-SF, Th.G. had a relatively good comprehension ability which was intact at the word level but it would decrease at the sentence level as the sentence complexity increased. His word-repetition ability was satisfactory, while his language production ability was limited to automized sequences. These characteristics, combined with his emotional stability and good motivation, suggest that Th.G. was a suitable candidate for the MIT intervention (e.g., Hurkmans et al., 2012; van der Meulen et al., 2012; Zumbansen et al., 2014a; Zumbansen and Tremblay, 2019). The baseline SPECT scan, however, revealed an extensive hypoperfused area on the left hemisphere, which extended beyond the left frontal lobe (Broca’s area) to a considerable proportion of the temporal lobe (Wernicke’s area) and the parietal lobe. Early studies report that for the MIT intervention to be successful, patients’ brain liaisons should not include more than half of Wernicke’s area or of the subcortical temporal isthmus area (Naeser and Helm-Estabrooks, 1985; Frumkin et al., 1990). Therefore, this finding, combined with the fact that the right hemisphere was found to have hypoperfused areas situated in the frontal lobe and the upper part of the parietal lobe and the temporal lobe, questioned the effectiveness of the MIT.

The discrepancy between the clinical assessment and the neuroimaging findings, as far as the patient’s comprehension ability is concerned, cannot be fully explained by the traditional “Wernicke-Geschwind” model (Broca, 1862; Wernicke, 1874). According to this model, Wernicke’s area is where the meaning of the word is extracted and, thus, severe impairments in this area should have resulted in dramatically limited language comprehension ability. This was not the case for our patient, though. Thus, we can either suggest that an adequate level of language comprehension had already been translocated from the hypofunctional left-sided Wernicke area to the corresponding area of the right hemisphere, or we can take into consideration more modern models, which might be able to better explain why a severe impairment on Wernicke’s area is not combined with severe difficulties in speech comprehension. One such model is the network-based dual stream model, suggested by Hickok and Poeppel (2004), according to which there are two pathways, the “dorsal” and the “ventral” streams, with the ventral one to be responsible for the speech signal processing and largely bilaterally organized from the temporal pole to the basal occipitotemporal cortex.

### Post-MIT Evaluation

After the completion of MIT, Th.G.’s ability to produce automatized, trained and non-trained speech improved impressively (see also Table 2). He became able to repeat whole sentences and to name items through oral descriptions or pictures (“Oral expression” section of BDAE). Moreover, he could use automatized sequences when requested and during simple social conversations, as indicated by the relevant subsections of BDAE. With the enrichment and the transformation of trained sentences, like “I want to drink coffee,” he became able to produce non-trained descriptions of his daily routine. The most impressive improvement, though, was apparent in his ability to use propositional speech, in order to describe a picture, such as that of “The cookie theft” picture, and, most importantly, in order to start and maintain a short conversation, about an issue relevant to his daily life. Thus, contrary to previous studies in which no findings regarding patients’ ability to produce non-trained items are reported (e.g., Goldfarb and Bader, 1979; Springer et al., 1993; Baker, 2000; Wilson et al., 2006; Hough, 2010), the present study offers clear descriptions of the patient’s propositional speech, which was dramatically improved after the completion of MIT. These results are even more impressive if we take into consideration the fact that our patient had very limited speech production ability for almost two and a half years before the beginning of the intervention. The aforementioned outcomes are in line with the goal of the MIT which is to restore generative language to patients with non-fluent aphasia (Albert et al., 1973; Sparks et al., 1974; Helm-Estabrooks et al., 1989; Sparks, 2008).

Interestingly, apart from his oral expression abilities, his comprehension and reading abilities also improved. In particular, he became able to follow more complex commands (with five different steps) and answer questions related to short passages. These results are consistent with Tabei et al. (2016) who also reported increased comprehension ability after the completion of the MIT intervention. As for the patient’s reading abilities, if we consider that almost half of the subsections of the Reading functional section also require and evaluate comprehension of words, sentences or paragraphs (Picture-word matching, Reading of sentences with comprehension, Comprehension of sentences and paragraph), we can suggest that part of the patient’s better performance is attributed to his improved comprehension ability. Moreover, if we adopt Tabei et al. (2016) claim, according to which the improvement in figure naming (oral expression) suggest an increase in the number of words retrieved from memory, and combine it with the claim that visual letter/number/word recognition is mainly based on the retrieval of the sound of the visual information (e.g., Vellutino et al., 2007), we can assume that our patient’s improvement in both functions is based on the fact that retrieval became easier and faster.

The aforementioned clinical profile was reinforced by our neuroimaging findings. Existing models support the concept that unilateral left-hemisphere lesions causing aphasia can lead to cortical inhibition of neighboring ipsilesional cortical areas, as well as to disinhibition of contralesional right-hemisphere homotopic areas connected via the corpus callosum (Shimizu et al., 2002). In patients with non-fluent aphasia, hemispheric involvement may be dynamic and change as a function of time: neither hemisphere is activated during the acute phase, the right hemisphere exhibits stronger involvement in the subacute phase and the left hemisphere appears to regain dominance in the chronic phase (Hillis, 2007). Our patient exhibited perceptible change between the baseline (first) and the post-MIT (second) scan that was visible at the periphery of the extensive hypoperfused stroke area of the left hemisphere. The perfusion improvement revealed by SPECT in this area suggests that this extensive hypoperfused cortical area surrounding the infarct core was not necrotic, but corresponded to chronic post-stroke impaired neuronal activity with subsequently compromised perfusion. The improved cortical regions after MIT comprised the rear sections of the parietal and the temporal lobe (location of the Wernicke’s area) and, to a lesser degree, the frontal lobe. Since Wernicke’s area has been mainly linked to language comprehension ability, these findings seem to be in line with the clinical improvement found in our patient, as far as comprehension is concerned. This significant improvement of left perilesional areas on SPECT after MIT is consistent with the notion of ipsilateral perilesional disinhibition, proposing that persistent recruitment of cortical areas in chronic aphasia can release the inhibitory input from the lesioned left-hemisphere cortex to nearby perilesional cortical networks, thus facilitating functional modifications characterized by increased activity of those perilesional areas and correlating with our patient’s clinical improvement (Hamilton et al., 2011).

On the other hand, the persistence of the spontaneously occurring increased activation of the right hemisphere beyond the subacute phase of a stroke has been considered as being suboptimal and largely maladaptive (Belin et al., 1996). The fact that our patient had not exhibited signs of chronic increased right-hemisphere activation before the therapeutic intervention, but quite the opposite, his baseline SPECT scan revealed chronic right-sided hypoperfusion in homotopic areas contralesional to the stroke, might account for his excellent clinical response to the intervention. Most of the areas exhibiting reduced cortical perfusion at baseline improved or even normalized as early as right after the MIT. If we assume that the Broca’s area on the right hemisphere corresponds to BA 44 and 45 (as on the left) and by analogy, the ipsilateral Wernicke’s region corresponds to BA 22, 39, and 40, then, the right-sided Broca increased from mildly hypoperfused in the baseline scan to almost normal in the second (post-MIT) scan and comparable perfusion increase was seen on the ipsilateral Wernicke’s area. This supports the idea that the functional architecture of right-hemisphere language areas may mirror that of the left hemisphere, suggesting that these right-sided networks may be modified beneficially with MIT training.

The connection between speech production and speech comprehension has been also found in previous studies (Awad et al., 2007; Stephens et al., 2010) and they are in line with the conclusion drawn by Schlaug et al. (2009), according to which, for an intervention to result in language production improvement, the temporal lobe must strengthen its connections with the frontal lobe in order to provide fast feedback mechanisms for vocal articulation and coupling for auditory-motor to take place (on the right hemisphere if the left has been affected). This observation, however, leaves also space for more contemporary neurological models to be compared to the traditional one which claims a clear division between production and comprehension processes. Some of these models suggest the existence of primary systems, related to the levels of the language (phonology, semantics, and syntax), which support both modalities (comprehension and production) (Patterson and Ralph, 1999; Menenti et al., 2011). Others, such as the dual-stream model proposed by Hickok and Poeppel (2004) or the one proposed by Mirman et al. (2015), suggest the existence of streams which can support functions essential to both speech production and speech comprehension tasks [for a selective review see also Nasios et al. (2019)].

As for the strong increase in the activation of the right hemisphere observed in our patient after the completion of MIT, this finding has been also reported in previous studies and it has been connected to the patients’ improved speech fluency (Schlaug et al., 2008, 2009; Zipse et al., 2012). In particular, Schlaug et al. (2008) reported an increase in the activation of the inferior frontal and temporal lobes, Schlaug et al. (2009) found an increase in the absolute number of fibers in the right arcuate fasciculus, whereas Zipse et al. (2012) demonstrated a profoundly increased activation of the right posterior middle frontal and the inferior frontal areas. These findings are in agreement with the meta-analysis of functional neuroimaging studies of chronic aphasia after stroke conducted by Turkeltaub et al. (2011), according to which, apart from left hemispheric areas or spared left hemispheric areas that are part of the language network, homotopic (to the left language areas) right hemispheric areas can also be recruited during recovery. The pattern, however, of both hemispheres to (be able to) support language production, has been also attested in several studies conducted on healthy participants (e.g., Guenther et al., 1998; Jeffries et al., 2003; Bohland and Guenther, 2006; Ozdemir et al., 2006), which conclude that, even though there is a tendency for greater left-lateralization during speaking, both hemispheres are activated. The predominant right hemisphere activation pattern found in patients after the administration of MIT has been attributed to MIT’s intonation, which leads to the reduction of the speech speed and the lengthening and the clearer chunking of the syllables, and to the left hand-tapping, which allows for timing predictability and engages the right-hemispheric sensorimotor network (Schlaug et al., 2008; Gordon et al., 2011; Stahl et al., 2011; Stahl and Kotz, 2014).

Therefore, the overall evaluation of our patient’s behavioral performance and brain perfusion scanning after the completion of MIT reveals that his language production was impressively improved after the completion of the intervention. The improvement was evident in both trained and untrained phrases, which consisted of up to five words and contained functional words in several mandatory language environments. Thus, the aim of the original MIT, which is for patients to be able to generate their own new phrases apart from using the trained ones (Albert et al., 1973; Sparks et al., 1974; Sparks, 2008), was accomplished. Regarding brain activity, as portrayed by SPECT, although the left perilesional areas were re-activated post-MIT, the perfusion of certain areas on the right hemisphere also improved considerably. This finding is also in line with the prediction made by the original MIT, according to which the right hemisphere is expected to take over the language functions of the damaged left hemisphere areas, after the completion of the MIT treatment (Albert et al., 1973; Sparks et al., 1974).

### Evaluation After 3 Months

Although the maintaining of the positive effects of an intervention is of great importance for both clinicians and patients, until today, to the best of our knowledge, there is only one study dealing with the long-term effects of MIT (Wilson et al., 2006). Wilson et al. (2006) administered thirty novel phrases of three experimental conditions: unrehearsed, rehearsed verbal production (repetition), and rehearsed verbal production with melody (MIT) to a 52 year-old, right-handed male. Comparison of performance at baseline, 1 week, and 5 weeks after therapy revealed that MIT rehearsed production was more durable. The results, however, could not be generalized, since the patient had been an amateur singer and composer for 25 years. Moreover, the investigation of the MIT effects was only focused on specific trained phrases and not on propositional speech. As the authors support, the 4 weeks of intervention might have been a short time for prepositional speech to appear. Lastly, the patient’s improvement was not supported and depicted by any neuroimaging technique.

The patient of the present study, however, was not a musician and, according to his performance in APT, his prosody comprehension abilities were poor. Therefore, the improvement found on his expressive language ability, after the completion of the MIT program, cannot be attributed to the patient’s exceptional understanding of the prosodic and musical elements, as could be suggested with the experienced musician who took part in the case study conducted by Wilson et al. (2006). The long-term evaluation revealed that the patient’s auditory comprehension and reading abilities remained quite stable, as was indicated by his performance on BDAE. Regarding the “Oral expression” functional section, we can notice that Th.G.’s performance was slightly better on word repetition and naming compared to the evaluation right after the completion of MIT. This finding comes in agreement with the patient’s family members’ claims, according to which, he became better and more interested in using new words instead of using complete sentences. As for the patient’s propositional speech, it appears to have slightly deteriorated, since his utterances consisted of fewer words compared to that just after the completion of MIT. This performance can be attributed to his refusal to revise some of the final steps of MIT hierarchy, with his family members acting as MIT administration assistants, and his refusal to drastically change the manner of communication he was using with his friends and family before MIT (use of single words, facial expressions and signs), since he was sure that everyone could understand him even if his utterances were not complete. On the other hand, it could also be the case that his family and friends did not know how to react and deal with the new condition (e.g., they kept giving him whatever he needed without him even having to ask for it). Considering, however, the patient’s inability to use more than two automatized or trained words before the treatment, his improvement was still impressive 3 months after the completion of the MIT program, even if his utterances were telegraphic and short.

The SPECT imaging at the 3-month follow-up confirmed that the Broca area on the left hemisphere remained heavily hypoperfused, since it was situated at the core of the post-stroke infarct. The perfusion of the Wernicke area improved significantly after the completion of the MIT and this improvement continued also between the second and the third scan. On the right hemisphere, the perfusion of the right-sided Broca area returned to normal in the third scan; most of this fair increase had already occurred during the second (post-MIT) scan and the remaining improvement occurred at 3 months. What was captivating, though, is the gradual increase of the perfusion observed on the right-sided Wernicke area (particularly area BA 39), which gradually increased from normal at baseline to hyperperfusion levels 3 months after MIT. Part of this increase occurred from the first to the second scan, though its largest fraction occurred from the second to the third scan. This hyperperfusion is consistent with an increased neuronal activity in these areas (Catafau, 2001) and could represent the activation of remote cortical centers related to language comprehension and speech as a result of the MIT intervention.

Therefore, in line with the claim of Sparks (2008), the overall results from both the BDAE and the SPECT scan revealed that not only were the MIT effects evident 3 months after the completion of the therapy, several brain regions had also improved further. This stability and improvement occurred without any further practice of the MIT steps or the application of another intervention program. It appears, though, that the patient’s (reduced) goals and will power limited the use of his newly acquired language production abilities.

## Conclusion

Even though our study was conducted on only one patient, MIT seems to be a promising intervention program for a long-term improvement of spontaneous speech. Patients with unilateral, left-hemisphere stroke, well-preserved comprehension abilities, restricted speech output and emotional stability are suitable candidates for the administration of MIT, even if they do not have any musical (formal or self-taught) educational background, or rarely listen to music and they are not good at the comprehension of the prosodic elements. MIT’s positive effects could appear even two and a half years after the stroke and remain stable, if not continuing to improve, for at least 3 months after the completion of the MIT intervention without any further revision of the MIT steps or any following application of an intervention program. It is advisable, however, for patients to continue receiving some kind of speech treatment after the completion of MIT, as it could help them stabilize the length of their utterances. Brain perfusion SPECT appears to provide subjective imaging evidence supporting the observed clinical improvement after the therapeutic intervention.

Even though we do not know the exact processes taking place through recovery, it seems that the traditional Wernicke-Geschwind model cannot account for the combination of the patients’ clinical and neuroimaging condition or for their recovery. Therefore, taking into account our patient’s good comprehension abilities–even though his Wernicke area was initially severely impaired–, as well as the connection found between speech production and speech comprehension and between the left and the right hemisphere, with regard to language functions, it is indicated that more recent brain and language models should be adopted.

## Data Availability Statement

The original contributions presented in the study are included in the article, further inquiries can be directed to the corresponding author/s.

## Ethics Statement

The study conformed with the Helsinki Declaration principles and was conducted in accordance with local legislation. The patients/participants provided their written informed consent to participate in this study. Written informed consent was obtained from the individual(s) for the publication of any potentially identifiable images or data included in this article.

## Author Contributions

MM conceived the idea, translated and adapted MIT to Greek, performed the intervention to the patient, and wrote the manuscript. AN recruited the patients and evaluated the patient’s language abilities before the intervention, immediately after the completion of the MIT, and 3 months later. GN evaluated the patient’s clinical and neurological condition. ST conducted the SPECT scans, analyzed the relevant results, and contributed to the writing of the manuscript. All authors contributed to manuscript revision, read, and approved the submitted version.

## Conflict of Interest

The authors declare that the research was conducted in the absence of any commercial or financial relationships that could be construed as a potential conflict of interest.
